# Predicting the Kinetic Properties Associated with Redox Imbalance after Oxidative Crisis in G6PD-Deficient Erythrocytes: A Simulation Study

**DOI:** 10.1155/2011/398945

**Published:** 2011-09-28

**Authors:** Hanae Shimo, Taiko Nishino, Masaru Tomita

**Affiliations:** ^1^Institute for Advanced Biosciences, Keio University, 403-1, Daihoji, Tsuruoka, Yamagata 997-0017, Japan; ^2^Department of Environment and Information Studies, Keio University, Endo 5322, Fujisawa, Kanagawa 252-8520, Japan; ^3^Systems Biology Program, Graduate School of Media and Governance, Keio University, Endo 5322, Fujisawa, Kanagawa 252-8520, Japan

## Abstract

It is well known that G6PD-deficient individuals are highly susceptible to oxidative stress. However, the differences in the degree of metabolic alterations among patients during an oxidative crisis have not been extensively studied. In this study, we applied mathematical modeling to assess the metabolic changes in erythrocytes of various G6PD-deficient patients during hydrogen peroxide- (H_2_O_2_-) induced perturbation and predict the kinetic properties that elicit redox imbalance after exposure to an oxidative agent. Simulation results showed a discrepancy in the ability to restore regular metabolite levels and redox homeostasis among patients. Two trends were observed in the response of redox status (GSH/GSSG) to oxidative stress, a mild decrease associated with slow recovery and a drastic decline associated with rapid recovery. The former was concluded to apply to patients with severe clinical symptoms. Low *V*_max_ and high *K*_mG6P_ of G6PD were shown to be kinetic properties that enhance consequent redox imbalance.

## 1. Introduction

Glucose-6-phosphate dehydrogenase (G6PD) deficiency, an X-chromosome linked genetic disorder, is the most prevalent mutation in humans affecting more than 400 million people worldwide [1–4]. It is characterized by the decreased activity of the G6PD enzyme, which is the central factor of the antioxidant defense system in erythrocytes (or RBCs). The enzyme is responsible for maintaining the high levels of reduced glutathione (GSH) and nicotine adenine dinucleotide phosphate (NADPH) that protect the cell from oxidative damage caused by harmful reactive oxygen species (ROS). 

As RBCs are unable to generate NADPH through other pathways [5, 6], G6PD-deficient RBCs lack the ability to tolerate excessive amounts of oxidative stress [7–9]. The most common clinical manifestation associated with G6PD deficiency is hemolytic anemia, which is generally triggered by the intake of oxidative drugs or foods [5, 10]. At times, the defect can result in complications such as kidney failure, severe neonatal jaundice, gallstones, and may require blood transfusion [2, 9–11].

The clinical consequences of drug-induced G6PD deficiency-related hemolysis depend on several factors including the kinetic properties of the G6PD variant. Commonly the diagnosis of patients is performed by a rapid fluorescent spot test of NADPH generation [12, 13] and a quantitative spectrophotometric assay of G6PD activity [10, 14]. Although these tests provide us with some perspective on the degree of severity of a patient's clinical symptoms, they are based on measurements of G6PD-specific activity when a patient is under normal conditions, and do not specifically predict the dynamic response of metabolite and enzyme activity levels in a patient's RBCs during the intake of oxidative agents. Moreover, carrying out the test during an acute hemolytic episode would give unreliable results, as the activity of the abnormal G6PD-deficient cells would be obscured by the large population of reticulocytes having relatively higher enzyme activities than the fully matured RBCs [9, 10, 15]. Consequently, the preclinical assessment of sensitivity to oxidative stress in individual G6PD-deficient patients through *in vivo* experiments remains a difficult task [16–18]. In this study, we apply mathematical modeling to assess the changes in antioxidant enzyme activities and redox status for various individuals with G6PD deficiency during acute exposure to an oxidative agent. The main objective of this paper is to study the differences in the metabolic alterations during drug-induced hemolytic conditions in G6PD-deficient patients and predict the kinetic properties that enhance the degree of redox imbalance after an oxidative crisis. 

Drug interaction in RBCs is known to produce hydrogen peroxide (H_2_O_2_) directly or by way of ROS [9]. We therefore expanded an existing model of human RBC metabolism [19] to include the component reactions of ROS production and their removal processes. By inputting parameters of G6PD-deficient patients into this model, we performed simulation experiments to assess the metabolic response during exposure to oxidative H_2_O_2_ in patients' RBCs. We designated indicators of redox imbalance (e.g., recovery time of GSH/GSSG) from resultant simulation data to evaluate the relationship between kinetic parameters for the G6PD reaction in the patient's RBC and the patient's ability to recover from the transient oxidative perturbation. The targets of our simulation included the RBCs of 10 G6PD-deficient patients based on real data (Table 1, [21]), and virtual G6PD-deficient patients composed of randomly chosen kinetic parameters. The results of these experiments predicted the kinetic properties of G6PD that make a patient suffer major alterations in redox homeostasis after an oxidative crisis. Our simulation results provide insights for improved patient-level treatment through the mathematical modeling and simulation of symptomatic situations, as well as a better understanding of the pathophysiology of G6PD deficiency. 

## 2. Materials and Methods

**Figure 1:**
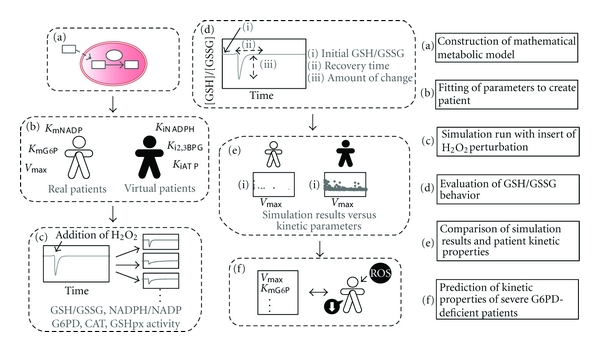
Schematic representation of the experimental workflow. (a) We first constructed a mathematical model of the human red blood cell (RBC) incorporating the antioxidant defense mechanism. (b) We then used published data from Jacobasch et al. [21] and fitted the patient-specific enzymatic kinetic parameters to build glucose-6-dehydrogenase phosphate (G6PD)-deficient patient models. Concurrently, we created virtual patient models. (c) The metabolic response of metabolite ratios and enzymatic activity to hydrogen peroxide (H_2_O_2_) perturbation was simulated. (d) Recovery time and amount of change of the ratio of reduced glutathione to glutathione ratio (GSH/GSSG) were used for evaluation of patient recovering ability. (e) The relationship between kinetic parameters and the indicators of redox imbalance were examined in both real and virtual patients. (f) The kinetic properties of G6PD-deficient patients with severe symptoms were predicted from analysis results.

The experimental workflow is presented schematically in Figure 1.

### 2.1. Representation of the Antioxidant System Using a Mathematical Model

A published model of human RBC metabolism [19] constructed in E-Cell System Environment Version 3 (E-Cell, [22, 23]) was used as a basis for the construction of our model of the oxidative stress removal mechanism in RBCs. The model is built up of major metabolic pathways including glycolysis, pentose phosphate pathway, adenine nucleotide metabolism, glutathione metabolism, several membrane transport systems, ion leakage processes, Na^+^/K^+^ pump, and binding reactions of metabolites to Mg^2+^ and hemoglobin. The rate equations are derived from previous experimental data and are described in the Supplementary Material (see Supplementary Material available online at doi: 10.1155/2011/398945).

**Figure 2:**
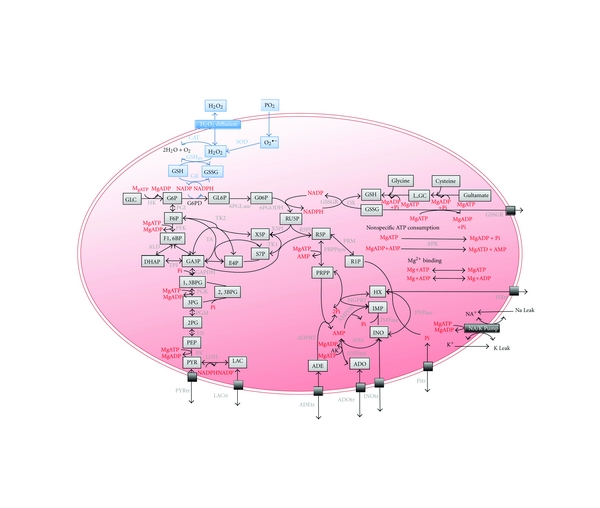
A pathway map of human red blood cell metabolism and antioxidant mechanism. The map is partially derived from Kinoshita et al. [19]. Gray boxes represent metabolites, and black boxes represent membrane transport or ion pump systems. Gray font indicates enzymes, and red font indicates the substrates or allosteric effectors of glycolytic enzymes. The blue region indicates the newly added pathway of reactive oxygen species (ROS) production and detoxification. Definitions of abbreviations for enzymes and metabolites can be found in the Supplementary Material.

In the developed model (Figure 2), we incorporated the diffusion of H_2_O_2_ into the RBC and the generation of O_2_^−^, as well as their removal processes catalyzed by the antioxidants catalase (CAT), superoxide dismutase (SOD), and glutathione peroxidase (GSHpx). Detailed descriptions of the enzymatic equations and initial parameter values can be viewed in the Supplementary Material.

### 2.2. Adjustment of Kinetic Parameters to Represent Individual Patients

The reaction catalyzed by the G6PD enzyme and the reaction rate (*ν*) are expressed by the following equations (G6P, glucose-6-phosphate; GL6P, gluconolactone-6-phosphate, 2,3BPG, 2,3-bisphosphoglycerate):

(1) $$\begin{array}{c} \text{G}6\text{P}+\text{NADP}\to \text{GL}6\text{P}+\text{NADPH},\\ \nu =\frac{V_{\max\;}\frac{\left[\text{NADP}\right]\left[\text{G}6\text{P}\right]}{K_{\text{mNADP}}K_{\text{mG}6\text{P}}}}{1+\frac{\left[\text{NADP}\right]}{K_{\text{mNADP}}}\left(1+\frac{\left[\text{G}6\text{P}\right]}{K_{\text{mG}6\text{P}}}\right)+\frac{\left[\text{NADPH}\right]}{K_{\text{iNADPH}}}+\frac{\left[\text{ATP}\right]}{K_{\text{iATP}}}+\frac{\left[2,\;3\text{BPG}\right]}{K_{\text{i}2,\;3\text{BPG}}}}, \end{array}$$

where *V*_max_ represents the maximum initial velocity of the reaction, and *K*_*m*_ and *K*_*i*_ stand for the concentration of substrate that produces half-maximal velocity and inhibitor constant, respectively. 

When building RBC models for simulation of individual patients, the parameters *V*_max_, *K*_mG6P_, *K*_mNADP_, *K*_iNADPH_, *K*_iATP_, and *K*_i2,3BPG_ were fit to the values specific to each patient. We first used parameter data from a previous study [21] to model 10 real G6PD-deficient patients and one healthy control. The hematological and kinetic parameters of the patients differed significantly (Table 1). Under the assumption that it would be difficult to predict the trends in the relationship between patients' kinetic parameters and their ability to restore steady state conditions after oxidative perturbation from the limited experimental data alone, we created virtual patients with these 6 parameters randomly chosen from a range of realistic values (Table 2), which ranged from the minimum to maximum values of the real patient parameter set (Table 1). Data for 500 virtual patients was obtained in each trial run.

### 2.3. Insertion of H_2_O_2_ Perturbation

To assess the dynamical response of the metabolic network of the RBCs during exposure to sudden high levels of oxidative stress, we first developed a model representing metabolic steady state and then added a perturbation of 0.1 mM of H_2_O_2_ (Figure 1(c)). The behavior over time of the ratio of reduced glutathione to oxidized glutathione (GSH/GSSG) and the ratio of reduced nicotinamide adenine dinucleotide phosphate to nicotinamide adenine dinucleotide phosphate (NADPH/NADP), as well as the enzymatic activity of G6PD, CAT, and GSHpx in each patient were investigated. We then calculated the GSH/GSSG at steady state, the time required for GSH/GSSG to return to the normal level after the perturbation, and the amount of change in GSH/GSSG during the perturbation to evaluate the potential of oxidative stress-induced redox imbalance (Figure 1(d), [24]). Henceforth, these indicators will be referred to as “initial GSH/GSSG,” “recovery time,” and “amount of change,” respectively. In the calculation of amount of change, we used the following equation:

(2) $$\text{Amount}\;\text{of}\;\text{change}=\frac{\left[\text{GSH}\right]/{\left[\text{GSSG}\right]}_{\text{ss}}-\left[\text{GSH}\right]/{\left[\text{GSSG}\right]}_{\min\;}}{\left[\text{GSH}\right]/{\left[\text{GSSG}\right]}_{\text{ss}}},$$

where [GSH]/[GSSG]_ss_ represents the initial GSH/GSSG, and [GSH]/[GSSG]_min_ represents the minimum level of GSH/GSSG during the observation period following the perturbation.

### 2.4. Analysis of Simulation Data

From the simulation results, we examined the relationship of initial GSH/GSSG, recovery time and amount of change with each kinetic parameter (*V*_max_, *K*_mG6P_, *K*_mNADP_, *K*_iNADPH_, *K*_iATP_, *K*_i2,3BPG_) of real G6PD patients and then performed the same analysis with the virtual patients. With these results, we plotted the values of the three indicators of redox imbalance in accordance to *V*_max_ and *K*_mG6P_ in a 3-dimensional (3D) plot to investigate the kinetic conditions that exhibit severe alterations in redox homeostasis when exposed to an oxidative agent.

## 3. Results

### 3.1. Metabolic Behavior of G6PD-Deficient RBCs during H_2_O_2_ Perturbation

The levels of GSH/GSSG and NADPH/NADP and the activity of G6PD, CAT, and GSHpx during sudden exposure to a high concentration of H_2_O_2_ were traced using the simulation model (Supplementary Figure  1). Substantial differences in the behavior of GSH/GSSG, NADPH/NADP, and G6PD activity in response to the input of H_2_O_2_ were observed among the patients. Patients 1, 4, and 8 showed the most distinctive results when compared to the control subject and other patients. The profiles of CAT and GSHpx activities were identical in all patients, but CAT activity increased to a greater degree. 

**Figure 3:**
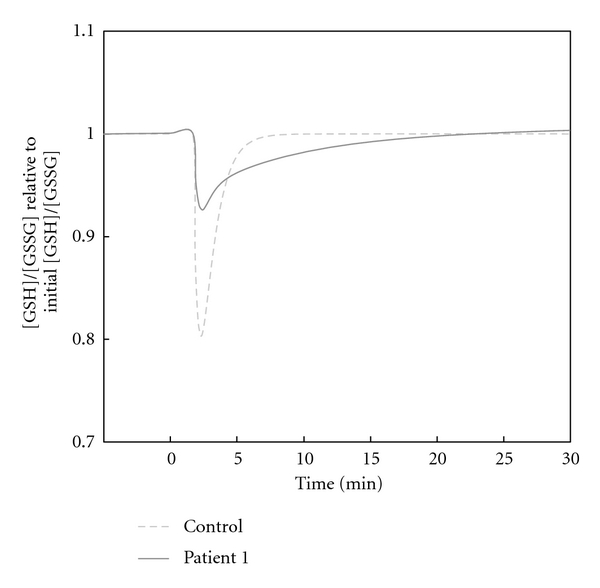
Comparison of the behavior of GSH/GSSG in the healthy control and patient 1 during H_2_O_2_ perturbation. 0.1 mM of H_2_O_2_ was added at 0 min, and the simulation was run for approximately 30 min. The *y*-axis represents the ratio of [GSH]/[GSSG] relative to the initial ratio, which is set as 1.0. The figure compares the healthy subject with the patient with the longest recovery time in the set of patients (i.e., patient 1).

A comparison of the redox status indicator GSH/GSSG in the healthy control and patient 1 is exhibited in Figure 3. The GSH/GSSG of patient 1 decreased only slightly during the oxidative perturbation; however, it took a relatively long time to return to the initial GSH/GSSG. In contrast, in the healthy control, GSH/GSSG levels dropped rapidly and returned to normal levels after a short period of time (Figure 3, Supplementary Figure 1).

### 3.2. Evaluation of GSH/GSSG Behavior

As mentioned previously, simulation results showed that the ability to recover from the sudden oxidative load varied among patients, in terms of recovery time and the amount of change. Therefore, in addition to initial values of GSH/GSSG, recovery time and amount of change were used to evaluate the degree of redox imbalance following oxidative stress perturbation in patients (Table 3). 

The resistance of RBCs to oxidative stress did not seem to correlate with a single kinetic parameter, such as *V*_max_ of the G6PD reaction as can be seen in patients 3, 4, and 8, which, despite having the same *V*_max_, presented distinctive recovery behavior. The healthy control had the highest initial GSH/GSSG and shortest recovery time, and patients 1, 4, and 8 who required long periods of time for recovery had the lowest initial GSH/GSSG (Table 3); patient 1 exhibited an abnormal recovery time in that the GSH/GSSG ratio did not return to the initial level during the period of observation. Overall, initial GSH/GSSG was proportional to the amount of change during the perturbation (correlation coefficient *R* = 0.99), and recovery time was inversely proportional to amount of change (*R* = 0.91, Supplementary Figure  2).

### 3.3. Effects of Kinetic Parameters on Antioxidant Capacity

Simulation results of steady state GSH/GSSG, recovery time, and amount of change were plotted against the *V*_max_, *K*_mG6P_, *K*_mNADP_, *K*_iNADPH_, *K*_iATP_, and *K*_i2,3BPG_ of real G6PD patients (Supplementary Figure  3); equivalent plots were constructed for virtual patients (Supplementary Figure  4). Of these 6 parameters, *V*_max_ and *K*_mG6P_ showed the highest association with the indicators of redox imbalance (Supplementary Figures  3 and  4); this trend was more obvious in the data for virtual patients than real patients because of the larger number of virtual patients. 

**Figure 4:**
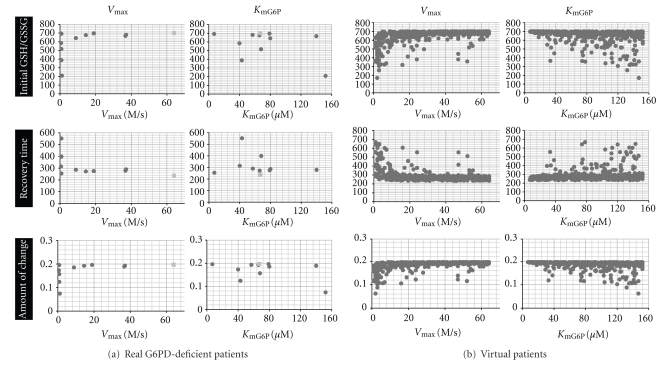
Comparison of G6PD kinetic parameters and steady state value, recovery time, and amount of change in GSH/GSSG in individuals. (a) The relationship between *V*_max_ and *K*_mG6P_ and the evaluation criteria in real patients (Table 1). Dark gray dots represent G6PD-deficient patients, and the light gray dots represents the control. (b) The relationship between *V*_max_ and *K*_mG6P_ and the evaluation criterions in virtual G6PD patients created from randomly selected parameters (Table 2). Recovery time denotes the time required for the GSH/GSSG to return to its initial value after perturbation, and amount of change is calculated by (2) in the text. Note that a few patients exhibiting abnormal recovery time (>30 min, 0.4% the of original number of patients) have been eliminated from the plot. The data displayed here were obtained from a single simulation run, which was representative of 15 simulation runs.

The distribution of plots for initial GSH/GSSG and amount of change were similar in both real and virtual patients (Figure 4). Most patients with high *V*_max_ or low *K*_mG6P_ appeared to have similarly high initial GSH/GSSG levels as well as similarly high amounts of change, whereas patients with low *V*_max_ and high *K*_mG6P_, showed a broad range of values for initial GSH/GSSG and amounts of change, and a greater proportion of patients with low values for these indicators. In the virtual patients, recovery time was inversely associated with *V*_max_ and positively associated with *K*_mG6P_ overall. Patients with high *V*_max_ or low *K*_mG6P_ tended to have short, similar times of recovery of around 200 s, whereas the recovery times in patients with low *V*_max_ or high *K*_mG6P_ were distributed between 200 s to 700 s. Several virtual patients did not conform to these trends: for example, patients with a high *V*_max_ but requiring a relatively long recovery time.

Simulation of virtual patient data was performed 15 times to ensure that there was no bias when determining the overall trend of the virtual patients. The degree of dispersion differed slightly between the trial runs; however, the traits given above were present in all results.

### 3.4. Analysis of the Relative Impact of  *V*_max_ and *K*_mG6P_ on the Determination of Symptom Severity

**Figure 5:**
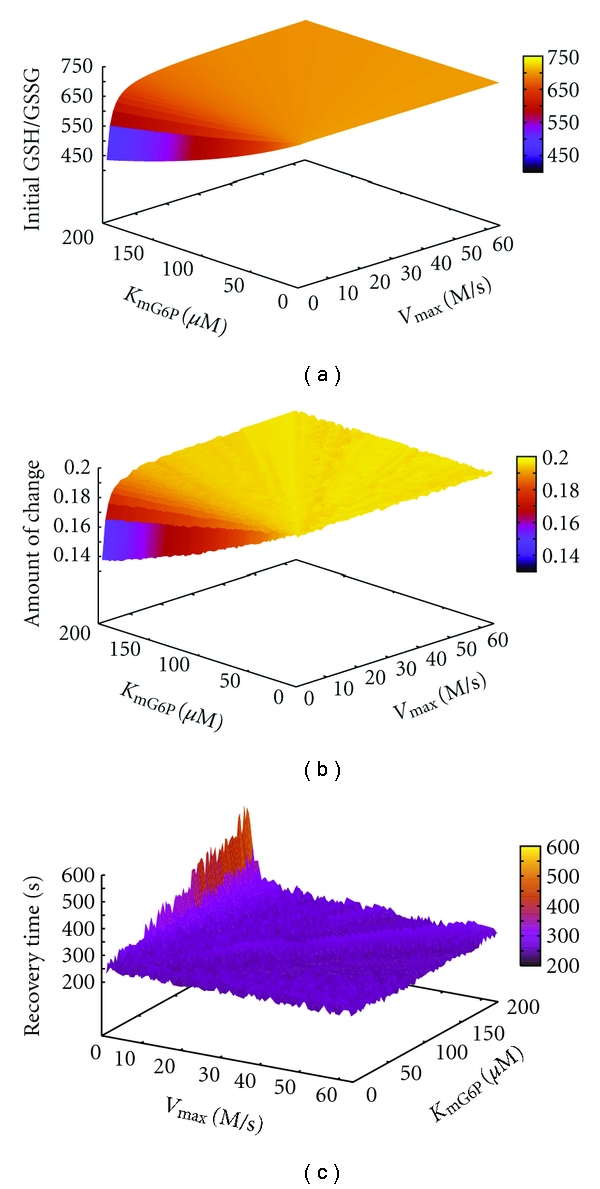
Correlation analysis of patient *V*_max_, *K*_mG6P_, and GSH/GSSG behavior. The figure shows GSH/GSSG behavior under fixed values for *K*_mNADP_, *K*_iNADPH_, *K*_iATP_, *K*_i2,3BPG_, and independent variables for *V*_max_ and *K*_mG6P_. *V*_max_ values within the range 0–64, and even values of *K*_mG6P_ within the range 0–200, were plotted against initial GSH/GSSG (left panel), recovery time (middle panel), and amount of change (right panel).

We then examined the relationship of *V*_max_ and *K*_mG6P_ with initial GSH/GSSG, recovery time, and amount of change by constructing a 3-dimensional plot (Figure 5). The distribution of the graphs for initial GSH/GSSG and amount of change were very similar. When *V*_max_ was low (*V*_max_ < 10), higher values of *K*_mG6P_ were associated with lower initial GSH/GSSG, lower amount of change, and longer recovery time.

## 4. Discussion

Deficiency in G6PD activity, and hence a disturbance in redox homeostasis, can lead to severe complications during the induction of an oxidative agent if not properly diagnosed (e.g., [25, 26]). Therefore, the preclinical assessment of the degree of metabolic dysfunction in a patient undergoing oxidative stress has been the focus of many past studies of G6PD deficiency [20, 27–29]. Although there have been several studies aiming at the mathematical representation and simulation of the metabolism in patients with G6PD deficiency [20, 30–32], no studies have sought to interpret the pathways for drug-induced ROS production or determine the kinetic properties that make G6PD-deficient RBCs suffer exceptionally from critical redox imbalance after acute exposure to an oxidative agent. In this study, we examined the metabolic changes in G6PD-deficient RBCs during exposure to H_2_O_2_ using a model that reproduced the oxidative-stress removal mechanism and evaluated how the alterations in redox homeostasis depend on the combination of kinetic parameters for enzymatic reactions in a patient's RBCs.

The time course metabolite data showed a decrease in GSH/GSSG and NADPH/NADP, and an increase in G6PD activity during the exposure to H_2_O_2_ in all patients, but each to a different extent. Such behavior accurately represented the antioxidant mechanism of H_2_O_2_ detoxification by G6PD, resulting in depletion of GSH, rapid production of GSSG, and a subsequent decrease of NADPH, as has been shown in previous experiments [33]. The similarity in CAT and GSHpx activity among patients, independent of the degree of deficiency of the G6PD enzyme, agrees with previous observations of normal levels of antioxidant enzymes such as CAT and GSHpx in G6PD-deficient patients, independent of their hemolytic crisis history [34, 35]. The increase in CAT and GSHpx activity in conditions where oxidative stress is sufficiently increased has also been shown in experimental data [34, 36]. Furthermore, CAT has been recognized to have a predominant role in the removal of ROS [37], which explains the relatively high activation of CAT seen in our simulation results. Although oxidative stress-induced changes were present in the levels of all metabolites and antioxidant enzyme activity, initial levels were restored shortly after the perturbation, supporting previous observations implying that RBC metabolism is not dramatically affected by the deficiency of G6PD enzyme activity [38]. 

 Schuster et al. [20] proposed that the severity of clinical symptoms in G6PD-deficient individuals could be assessed based on the maximal flux stimulated by an oxidative substance and upper oxidative load boundaries. However, we focused on the time-course restoration of GSH/GSSG; a redox couple that has been widely accepted as a reliable index in the estimation of cellular redox status [39]. Simulation results predicted the presence of two types of behavior in the recovery of GSH/GSSG among patients. Patients 1, 4, and 8 showed opposite traits to those of the other patients, with a change in GSH/GSSG that was relatively low in magnitude, but required long periods of time for recovery. In the control patient, GSH/GSSG dropped rapidly but recovered quickly, suggesting flexibility in the adaption to sudden change. 

**Figure 6:**
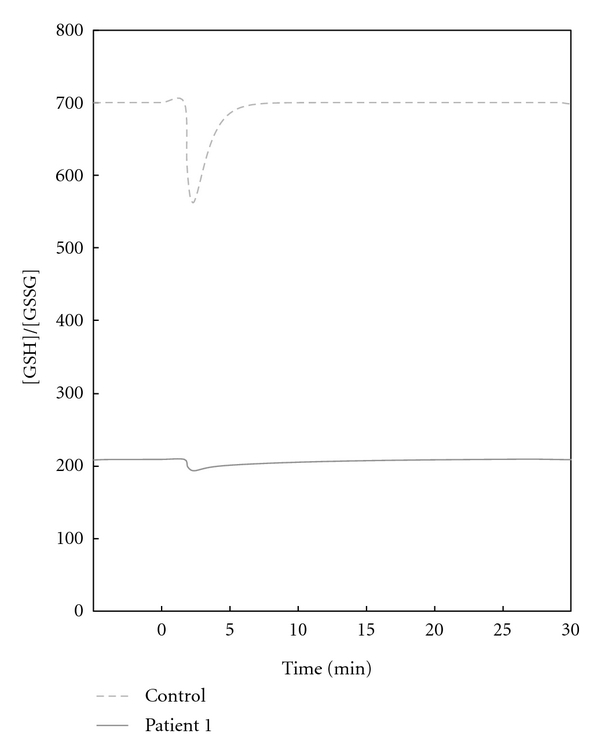
Comparison of time course GSH/GSSG behavior in healthy subject (above) and patient 1 (below). GSH/GSSG is given as the concentration of GSH relative to GSSG at steady state.

One might expect that oxidative stress would decrease GSH/GSSG with greater magnitude in patients with severe G6PD deficiency than those who are relatively healthy; however, in this case we found that GSH/GSSG greatlydeclined in the healthy control. The indication of sensitivity to stress being greater in the control patient in terms of magnitude was similar to the result of a previous report made by Arese et al. [27], who showed that oxidative divicine strongly stimulated the pentose phosphate pathway in normal RBCs, but hardly affected the metabolism in deficient cells. We speculated that the reason for the minimal amount of change of GSH/GSSG in patients 1, 4, and 8 is the patients' initial GSH/GSSG (Table 3, Figure 6). Patients 1, 4, and 8 displayed not only the longest recovery times but also the lowest initial GSH/GSSG. It is plausible that in RBCs where the initial GSH/GSSG is very low, if GSH/GSSG were to decrease greatly, the RBC could not function. We consider that the antioxidant mechanisms in the RBC may act to lessen the magnitude of GSH/GSSG reduction in such situations, and the recovery time may be lengthened as a result. Also from the accepted knowledge that cells with normal G6PD activity are highly capable of maintaining a steady GSH/GSSG [40], and redox imbalance in the RBC triggers the oxidant damage of lipids and proteins of the membrane and thus destruction of the RBC [40, 41], we propose that patients exhibiting a long recovery time of GSH/GSSG (i.e., severe redox imbalance) are the most severe and susceptible to hemolytic crises. Hence it can be concluded that patients 1, 4, and 8, which exhibit long recovery time and the small initial GSH/GSSG; opposite qualities of the healthy control, are the utmost severe G6PD-deficient patients who suffer severe clinical symptoms during the intake of an oxidative substance. Although there are no clinical descriptions of patients 4 and 8, it has been noted that patient 1 was diagnosed with severe chronic nonspherocytic hemolytic anemia [20], supporting our classification of patient 1 as a severe patient. 

The expansion of simulation to include virtual patients with parameters randomly selected from a range of existing values provided a broader view of the traits in patients, simplifying the discovery of trends and enabling the prediction of susceptibility to oxidative stressin patients from their kinetic parameters (Supplementary Figure  4). The indicators of redox imbalance in patients were independent of the kinetic parameters in most graphs; however, those with *V*_max_ and *K*_mG6P_ as the *x*-axis, showed sign of a trait (Figure 4). Although patients with high *V*_max_ (*V*_max_ > 40) and those with low *K*_mG6P_ (*K*_mG6P_ < 40) showed similarly high recovery abilities, as *V*_max_ decreased and *K*_mG6P_ increased, the proportion of patients with low initial GSH/GSSG, long recovery time, and small amounts of change increased. These findings suggest that low *V*_max_ and high *K*_mG6P_ enhance the likelihood of a patient having severe clinical symptoms during intake of an oxidative agent. From our observation that there were patients exhibiting short recovery time even in the low-*V*_max_ and high-*K*_mG6P_ groups, we can assume that the severity of symptoms cannot be determined by one single kinetic parameter. Also during simulation, a few virtual patients (not shown in Figure 4) exhibited abnormally long recovery times that were longer than the test period, and so had a similar recovery profile to that of patient 1. Because we have linked a mild decrease of GSH/GSSG and long recovery time with severe disorder in redox homeostasis in patients following a hemolytic crisis, we assume that these virtual patients correspond to the most severe patients.

The results of the kinetic parameter analysis of virtual patients using a 3D image plot (Figure 5) led us to predict that in the subset of patients with low *V*_max_, increases in *K*_mG6P_ will be associated with increases in the severity of the patients' symptoms. These results agree with past proposals of low *V*_max_ and high *K*_mG6P_ association with clinical severity of G6PD patients [16, 42, 43]. In fact, many patients with such properties have been classified into the most severe class of G6PD deficiency (i.e., Class I [5]) during diagnosis [16]. However, in this study, there were numerous virtual patients with low *V*_max_ that showed high capabilities of detoxifying oxidative stress. This contradicts the principal classification of G6PD-deficient patients [5], which mainly groups patients according to the *V*_max_ of the G6PD enzyme. The method of randomly choosing parameters, used here, is likely responsible for this disagreement. In this process, a combination of parameters that are not biologically feasible may have been produced, resulting in abnormal results. For example, an earlier work proposed that a low affinity to NADP may be linked to low inhibition by NADPH, ATP, and 2,3BPG [21]; however, in our simulation method, this was not taken in account.

## 5. Conclusion

In conclusion, we successfully constructed a dynamic model to represent the metabolic alterations in G6PD-deficient RBCs during exposure to oxidative stress induced by H_2_O_2_. We conclude that low initial GSH/GSSG is linked with mild decrease and delay in the recovery of the steady state GSH/GSSG after induction of an oxidative agent, and patients having this trait are susceptible to severe clinical symptoms. Furthermore, we conclude that recovering abilities do not rely on a single kinetic parameter such as *V*_max_. Our simulation results predicted a higher redox imbalance following oxidative damage in patients with low *V*_max_ and high *K*_mG6P_. In a disorder with more than 400 biochemical variants exhibiting distinct clinical manifestations [3, 29], in which the quick screening of a large number of patients requires complicated and expensive tests that may give abnormal results [13, 44, 45], *in silico* experiments such as those presented here will likely be significant for the accessible prediction of severity in pathophysiological conditions in patients without actual *in vivo *experimental procedures. We anticipate that our studies will also provide novel insights that will facilitate the implementation of mathematical analysis and simulation for better patient diagnosis in the future.

## Supplementary Material

The supplementary material contains additional simulation results that were used to construct Figures 3, 4, and 6, and make an assumption of the relationship between the indicators of redox imbalance. It also includes a detailed description of the E-Cell human erythrocyte model, involving the initial steady state concentrations of substrates and the kinetic equations and parameters used for simulation.

## Figures and Tables

**Table 1:** Hematological and kinetic parameters of G6PD-deficient patients (obtained from [19, 20]).

Patient	Reticulocytes (%)	Half-life (days)	*V*_max_ (M/s)	*K*_mG6P_ (*μ*M)	*K*_mNADP_ (*μ*M)	*K*_iNADP_ (*μ*M)	*K*_iATP_ (*μ*M)	*K*_i2,3BPG_ (*μ*M)
Patient 1	40	2.5	1.1	152	3.8	0.62	180	520
Patient 2	2	—	36.7	140	115	33.6	4687	8515
Patient 3	2	22.5	0.8	7	4.1	8.9	952	1071
Patient 4	22	—	0.8	43	155	56	11000	35000
Patient 5	2	20.0	37.1	57	30.5	3.7	5016	5507
Patient 6	15	7.2	14.5	66	3.5	1.1	212	532
Patient 7	10	—	8.9	80	3.6	1	125	586
Patient 8	4	—	0.8	68	1.4	0.9	500	2000
Patient 9	30	5.0	0.6	40	4.8	6.9	314	3784
Patient 10	15	—	18.9	79	3	4.1	407	2200

Control	1	27	64	67	3.7	3.1	749	2289

**Table 2:** *V*_max_ and kinetic constants of enzymatic reactions in virtual patients' RBCs.

Proband	Range of randomly chosen values
*V*_max _	1–64 (M/s)
*K*_mG6P_	7–152 (*μ*M)
*K*_mNADP_	3–155 (*μ*M)
*K*_iNADPH_	1–56 (*μ*M)
*K*_IATP_	125–11000 (*μ*M)
*K*_i2,3BPG_	520–35000 (*μ*M)

**Table 3:** GSH/GSSG behavior of G6PD-deficient patients.

Proband	*V*_max_ (M/s)	Initial [GSH]/[GSSG]	Recovery time (s)	Amount of change (ratio)
Patient 1	1.1	209.04	—	0.07
Patient 2	36.7	666.76	279.21	0.19
Patient 3	0.8	692.25	254.31	0.20
Patient 4	0.8	388.00	552.41	0.12
Patient 5	37.1	680.83	289.74	0.19
Patient 6	14.5	675.73	272.29	0.19
Patient 7	8.9	642.44	286.27	0.19
Patient 8	0.8	515.93	398.94	0.16
Patient 9	0.6	585.01	314.17	0.17
Patient 10	18.9	696.04	275.23	0.20

Control	64	700.38	235.52	0.20
