# Supplementary figures and images for: Predicting the Kinetic Properties Associated with Redox Imbalance after Oxidative Crisis in G6PD-Deficient Erythrocytes: A Simulation Study
Source: Adv Hematol. 2011 Sep 28;2011:398945. doi: 10.1155/2011/398945 (PMC3184397; doi:10.1155/2011/398945)

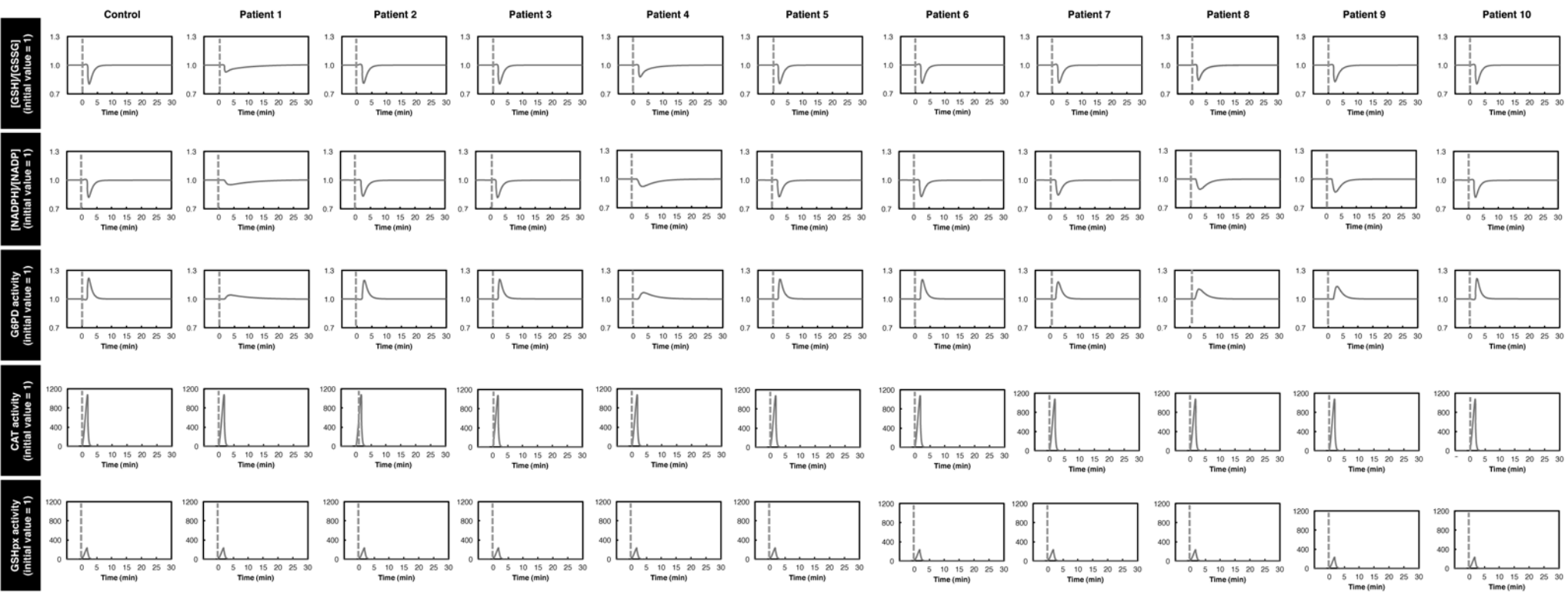

Supplement: Supplementary file 1 — The supplementary material contains additional simulation results that were used to construct Figures 3, 4, and 6, and make an assumption of the relationship between the indicators of redox imbalance. It also includes a detailed description of the E-Cell human erythrocyte model, involving the initial steady state concentrations of substrates and the kinetic equations and parameters used for simulation. [file 398945.f1.pdf]

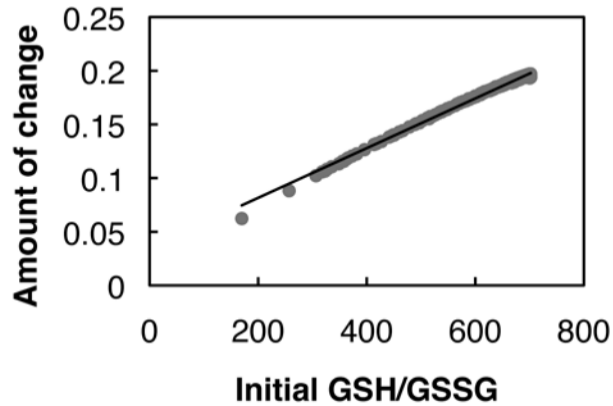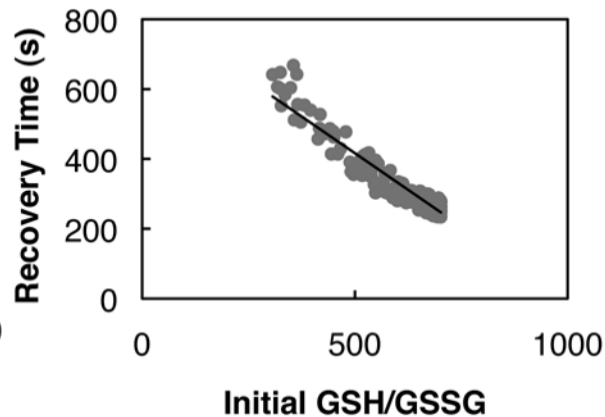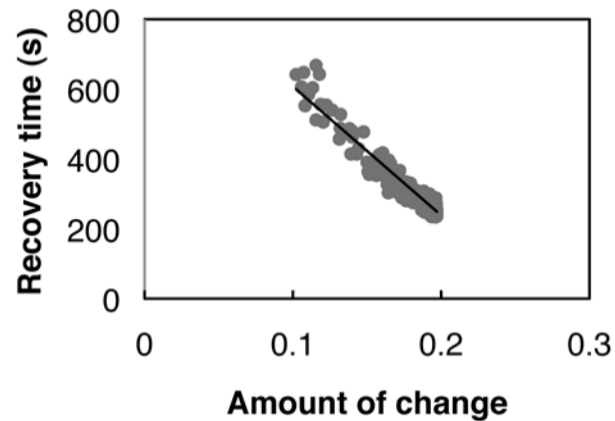

Supplement: Supplementary file 2 [file 398945.f2.pdf]

Initial GSH/GSSG

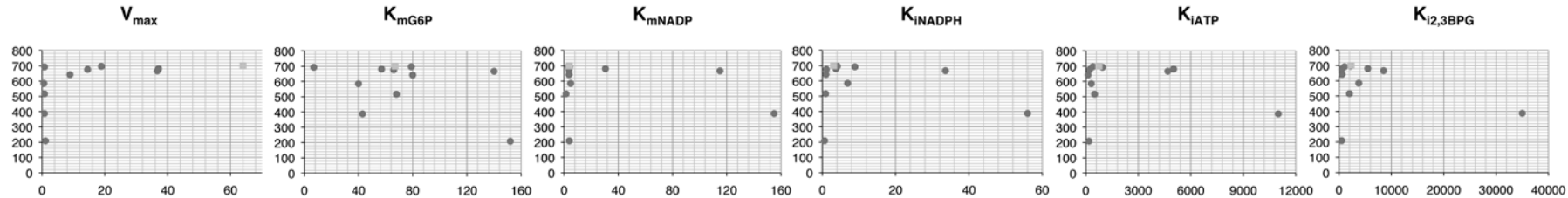

Recovery Time

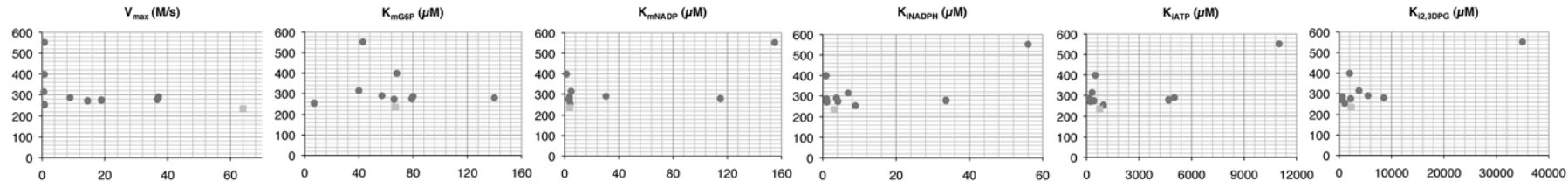

Amount of change

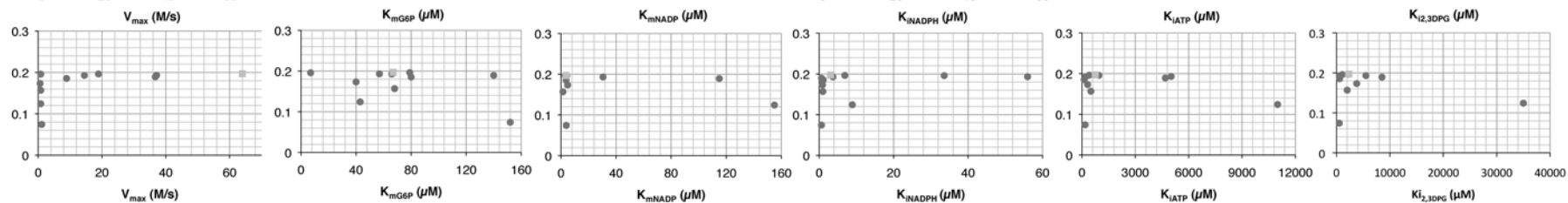

Supplement: Supplementary file 3 [file 398945.f3.pdf]

Initial GSH/GSSG

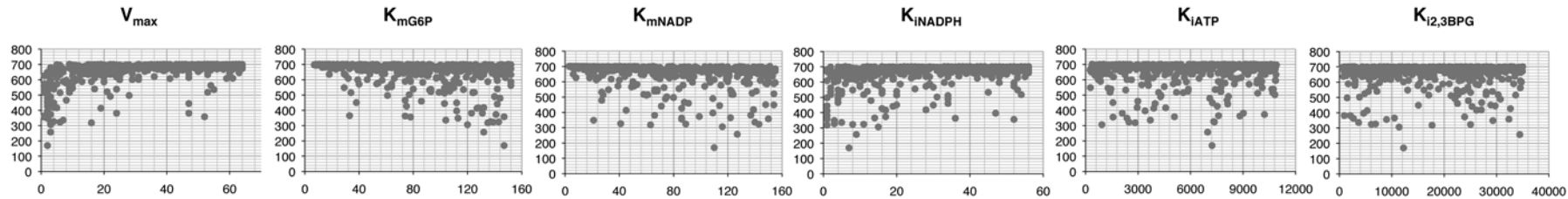

Recovery Time

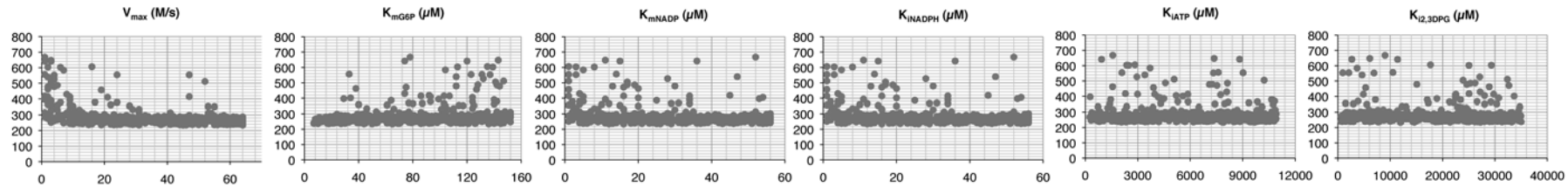

Amount of change

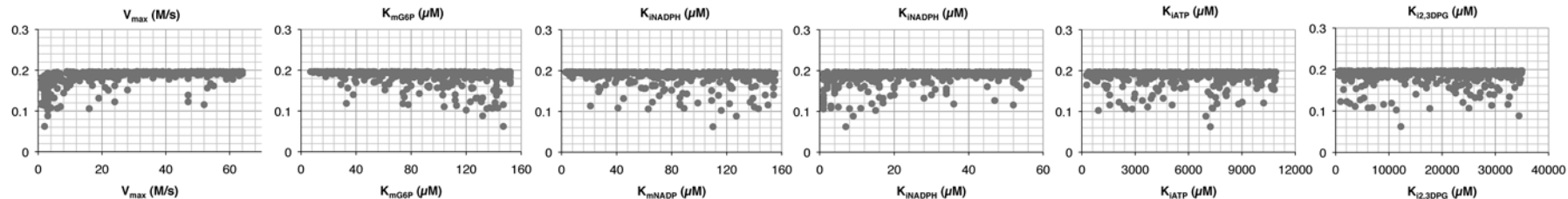

Supplement: Supplementary file 4 [file 398945.f4.pdf]
